# PTEN R130Q Papillary Tumor of the Pineal Region (PTPR) with Chromosome 10 Loss Successfully Treated with Everolimus: A Case Report

**DOI:** 10.3390/curroncol28020121

**Published:** 2021-03-20

**Authors:** Hazem I. Assi, Rasha T. Kakati, Juliett Berro, Ibrahim Saikali, Bassem Youssef, Roula Hourany, Ibrahim Alameh, Abeer Tabbarah, Jessica Khoury, Houssein Darwish, Saada Alame

**Affiliations:** 1 Division of Hematology & Oncology, Naef K. Basile Cancer Institute, American University of Beirut Medical Center, Cairo Street, Beirut P.O. Box 11-0236, Lebanon; jb78@aub.edu.lb (J.B.); jk93@aub.edu.lb (J.K.); 2 Faculty of Medicine, American University of Beirut, Bliss Street, Beirut P.O. Box 11-0236, Lebanon; rtk12@mail.aub.edu; 3 Division of Neurosurgery, Lebanese American University Medical Center, Beirut P.O. Box 11-3288, Lebanon; ibrahim.saikali@laumcrh.com; 4 Division of Radiation Oncology, American University of Beirut Medical Center, Cairo Street, Beirut P.O. Box 11-0236, Lebanon; by04@aub.edu.lb; 5 Division of Diagnostic Radiology, American University of Beirut Medical Center, Bliss Street, Beirut P.O. Box 11-0236, Lebanon; rh64@aub.edu.lb; 6 Department of Internal Medicine, American University of Beirut Medical Center, Cairo Street, Beirut P.O. Box 11-0236, Lebanon; ia84@aub.edu.lb; 7 Department of Pathology and Laboratory Medicine, American University of Beirut Medical Center, Cairo Street, Beirut P.O. Box 11-0236, Lebanon; at30@aub.edu.lb; 8 Division of Neurosurgery, American University of Beirut Medical Center, Cairo Street, Beirut P.O. Box 11-0236, Lebanon; had06@aub.edu.lb; 9 Department of Pediatric Neurology, Faculty of Medicine, Lebanese University, Beirut P.O. Box 6573/14, Lebanon; saadaalame@gmail.com

**Keywords:** papillary tumors of the pineal region, pineal parenchymal tumor, everolimus, PTEN, case report

## Abstract

Papillary tumors of the pineal region (PTPR) can be observed among adults with poor prognosis and high recurrence rates. Standards of therapy involve total surgical excision along with radiation therapy, with no promising prospects for primary adjuvant chemotherapy, as long-term treatment options have not been explored. Chromosome 10 loss is characteristic of PTPR, and *PTEN* gene alterations are frequently encountered in a wide range of human cancers and may be treated with mTORC1 inhibitors such as everolimus. In parallel, there are no reports of treating PTPR with everolimus alone as a monopharmacotherapy. We report the case of a patient diagnosed with PTPR (grade III) characterized by a *PTEN R130Q* alteration with chromosome 10 loss that was treated with everolimus pharmacotherapy alone, resulting in an asymptomatic course and tumor regression, a rare yet notable phenomenon not described in the literature so far with potential to alter the management approach to patients with PTPR.

## 1. Introduction

Papillary tumors of the pineal region (PTPR) are rare pineal parenchymal tumors with a 1% prevalence among intra-cranial tumors in adults and which more commonly arise in children [1]. PTPR arise from epithelial and interstitial cells of the pineal region, with expression of neuron-specific enolase, cytokeratins, S-100 protein, Glial Fibrillary Acidic Protein (GFAP), and vimentin, and were first classified as either grade II or grade III by the WHO in 2007 [1,2,3]. Although data are limited on the prognosis, surgical resection and increased mitotic and proliferative indices are prognostic indicators for PTPR [2]. Gross total resection has been established as the definitive treatment along with radiotherapy; nevertheless, the clinical course of PTPR is characterized by very high recurrence rates, with a cohort study demonstrating recurrence after a median time of 29 months in 56% of patients [1,2,3,4]. Few cases have been reported of PTPR treated with chemotherapy; nevertheless, a large study demonstrated that chemotherapy does not seem to influence overall survival or PFS [5]. Outcomes remain poor and fatal in patients with PTPR, with 5-year progression-free survival (PFS) at 27% [3,5,6]. Whether surgery, radiotherapy, or chemotherapy is used alone or in combination, studies suggest that PTPR is inevitably prone to recurrence [3]. As such, the necessity of novel therapeutic targets to prolong remission and PFS comes into light.

Molecular studies have demonstrated that over 75% of PTPR are characterized by loss of chromosome 10 [5]. The phosphatase and tensin homologue deleted on chromosome 10 (*PTEN*) gene located on chromosome 10q23 is a tumor suppressor gene and is inactivated in many brain tumors, which are frequently encountered in PTPR [7,8]. The PTEN protein regulates the phosphatidylinositol 3-kinase (PI3K) pathway, which produces phosphatidylinositol (3,4,5)-trisphosphate (PIP3), ultimately leading to the activation of AKT, a serine threonine kinase; AKT prevents the transcription of cell cycle regulators and activates mTORC1, which activates protein synthesis machinery, promoting cell growth, survival, and angiogenesis [9,10]. Cytoplasmic PTEN is a lipid phosphatase that directly inhibits PIP3K, thus inhibiting downstream pathways implicated in cancer cell proliferation and survival; nuclear PTEN promotes chromosome stability and regulates DNA double-strand break repair, further preventing cancer cell survival [9]. Loss of PTEN phosphatase activity results in constitutive overactivation of the PI3K/AKT/mTOR pathway to drive cancer cell propagation [9].

In patients with PTEN loss, considerations arise for targeting downstream signaling via mTORC1 inhibitors such as everolimus, reducing the activity of the downstream effectors and inhibiting cancer cell proliferation and survival, as well as angiogenesis [7]. Studies suggest the potential efficacy of everolimus in controlling the growth of *PTEN*-deficient cancers [9]. Metastatic prostate cancer and advanced pancreatic neuroendocrine tumors associated with *PTEN* loss have exhibited increased PFS with everolimus treatment combined with other drugs; however, this has not been thoroughly investigated in PTPR [3,8,9]. Only one report in an adult patient with *PTEN*-mutated PTPR and a dysregulated PI3K/AKT/mTOR pathway described a durable response to everolimus within 19 months, however only after a course of temozolomide chemotherapy [3,11]. In addition to the lack of consensus regarding therapeutic strategies and prognosis, to date no studies or reports have described treatment with everolimus as a monopharmacotherapy in adults with PTPR to investigate this drug’s impact [11].

We describe a case of recurrent intracranial anaplastic PTPR characterized by a *PTEN* R130Q alteration with chromosome 10 loss that was treated with everolimus as a monopharmacotherapy after surgical excision and radiotherapy, resulting in an asymptomatic course and tumor regression, a rare phenomenon not described in the literature so far.

## 2. Case Presentation

In March 2017, a 25-year-old male diagnosed with pineal anaplastic ependymoma (WHO grade III) at another institution in 2013 presented to our tertiary healthcare center to follow up his disease status after complete resection via craniotomy followed by adjuvant radiotherapy, with 2 years of stable disease shown on Magnetic Resonance Imaging (MRI)s.

However, in August 2016, an MRI showed local recurrence and disease progression, with five lesions with cystic and solid enhancement noted upon imaging; three lesions were located in the left and right cerebellar hemispheres, with the largest diameter measuring 2.5 cm, while two lesions were located in the tectal–pineal region, with the largest measuring 1.4 cm. His physician at another institution recommended re-irradiation at this point, after which the patient then presented to our institution in April 2017 to request a second opinion, reporting severe, throbbing headaches of intermittent frequency and associated with sonophobia and photophobia and not relieved by painkillers, as well as mild imbalance during tandem gait in a physical exam. He had been maintained on daily dexamethasone (1 mg) for vasogenic edema and relief of headaches since the surgical resection. Repeat brain MRIs showed disease progression in both the pineal and cerebellar masses, with extension into the left third ventricle.

Surgery was not an option at this point; a multidisciplinary team recommended comprehensive genomic profiling (CGP) and opted for a FoundationOne^®^ genomic test (Foundation Medicine, Cambrige, MA, USA), which revealed *PTEN* loss with *R130Q* alteration and a single copy number deletion of chromosome 10, a characteristic of Papillary Tumor of the Pineal Region (PTPR), after which a definitive diagnosis of PTPR was established. A multidisciplinary discussion in June 2017 culminated in starting treatment with 10 mg of everolimus (Afinitor^®^, Novartis Pharma GmbH, Nuremberg, Germany) and continuing with 1 mg of dexamethasone for headaches.

An MRI done after 2 months of treatment with everolimus in August 2017 showed increases in the sizes of the cerebellar masses, with progressive perilesional vasogenic edema with mild obstructive hydrocephalus, despite no change in the pineal lesions, until September 2017 when the pineal mass increased in size.

**Figure 1:**
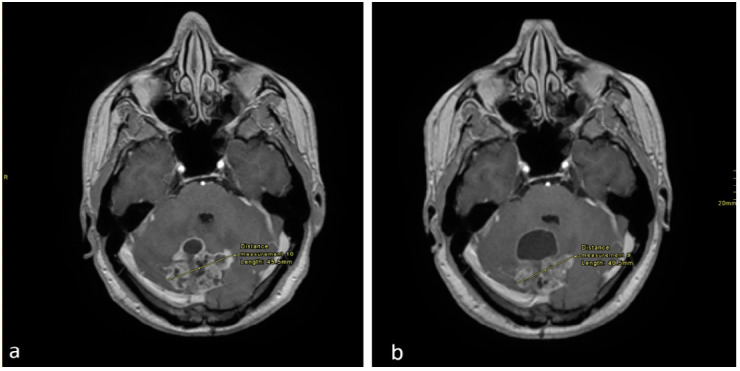
Axial enhanced T1 weighted images of the brain at the level of the cerebellum (**a**), performed in September 2017 and (**b**) in December 2017. There is an interval decrease in the size of the solid enhancing mass, however there is an increase in the anterior cystic component.

**Figure 2:**
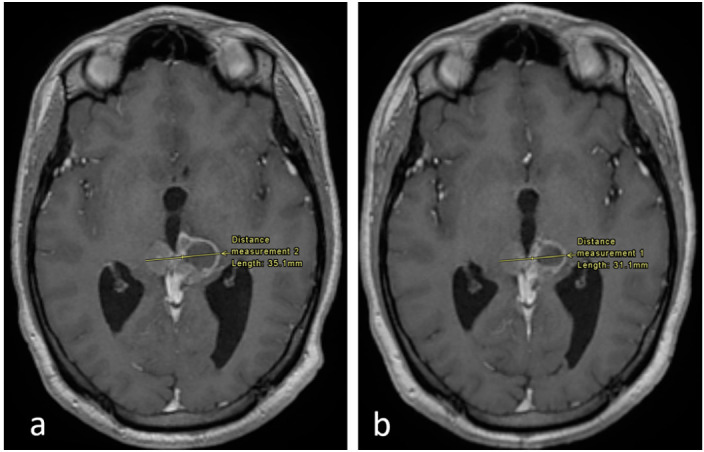
Axial enhanced T1 weighted images of the brain at the level of the pineal region. (**a**) Imaging performed in September 2017 and (**b**) in December 2017. There is an interval decrease in the size of the tumor, as shown.

Follow-up MRIs after 6 and 9 months of treatment in December 2017 and March 2018 revealed reductions in the solid components of both the cerebellar and pineal masses, despite an increase in the cystic component and persistent hydrocephalus (Figure 1 and Figure 2). Since the patient was asymptomatic, dexamethasone was discontinued at that point.

A maximal response to everolimus was achieved. In an attempt to maximize response and reduce tumor burden for the patient while having reached the maximum tolerable dose, a multidisciplinary team meeting yielded the decision to proceed with a suboccipital posterior fossa craniotomy with endoscopic third ventriculotomy in June 2018 to resect the cerebellar lesions, after which the patient experienced no complications and continued everolimus. An official pathology report of the resected cerebellar mass revealed monomorphic cells with scattered larger atypical cells arranged in perivascular pseudo-rosettes and true rosettes, in addition to necrosis and microvascular proliferation. The tumor intensely stained GFAP in the cytoplasmic processes, especially in perivascular tumor cells, in addition to cytokeratin 8/18.

In July 2018, follow-up MRI showed regression in the size of the pineal tumor. To further maximize response, and in-line with the multidisciplinary decision, the patient then received radiotherapy for 2 months, after which MRI showed further regression, followed by stability in size noted in September 2019.

MRI done in January 2020 then showed an increase in the size of the pineal gland tumor, which then remained stable until June 2020, after which surgical resection was performed. At the patient’s last follow-up in January 2021, he was completely asymptomatic while being maintained on everolimus. The patient provided fully informed, voluntary, and written consent to publish his data.

## 3. Discussion

**Figure 3:**
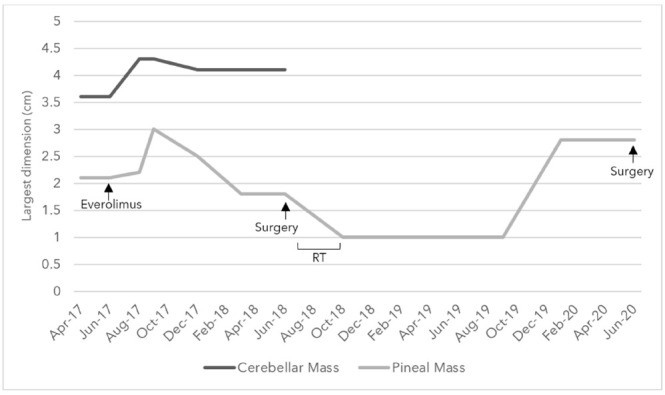
Changes in the size of the largest diameter of the cerebellar and pineal solid components in centimeters as noted in Magnetic Resonance Imaging (MRI) over the course of everolimus, surgery, and radiotherapy (RT) treatments between April 2017 and June 2020.

An adult patient diagnosed with anaplastic ependymoma (WHO grade III) presented to our healthcare center in 2017, with disease progression in cerebellar and pineal masses noted on MRI after complete resection, radiotherapy, and stable disease for 2 years. Next-generation sequencing revealed a *PTEN P130Q* alteration with chromosome 10 loss, and the patient was, thus, definitively diagnosed with PTPR (grade III) and started on 10 mg everolimus in June 2017. Slight disease progression was then demonstrated, followed by mild reduction in size then subsequent stability before further natural relapse, after which surgery was done to maximize response and quality of life after reaching the maximum tolerated dose of everolimus (Figure 3).

Histologically, rosettes were observed in addition to positivity for GFAP and cytokeratin. Several histological similarities make it difficult to discern a definitive diagnosis of PTPR in light of a differential diagnosis of an ependymoma, as reported in the literature [3]. However, chromosome 10 loss is a characteristic hallmark of PTPR that is not seen in ependymomas and which tipped our diagnosis [12]. This shows that CGP is key to diagnosis, although immunohistochemistry is an important component in diagnosing either tumor type.

Considering that there are no approved therapies specific to *PTEN P130Q* alterations of this tumor type, the healthcare team chose to treat him with everolimus, although this is rarely used for PTPR. The patient experienced a smooth clinical course with no adverse effects from treatment and has been maintained on everolimus for 43 months now, with a completely asymptomatic course throughout, suggesting notable implications for patient quality of life. Control and an overall trend of tumor regression were attained over 27 continuous months on everolimus. In light of the nature of recurrent relapses in PTPR, the value of prolonged remission is essential; everolimus is deemed an essential therapeutic consideration for a prolonged window of asymptomatic disease control, with implications for prolonged PFS and improved quality of life.

This is the first case to report the treatment of adult PTPR (grade III) with response to everolimus as a monopharmacotherapy, with attainment of remission within less than one year of treatment with everolimus and overall control for over two years, the longest follow-up duration noted for such a case in the literature. Accordingly, there remains a necessity for future studies with large sample sizes to further validate the therapeutic effects of everolimus in adult patients with PTPR, and we recommend further investigation.

## 4. Conclusions

Everolimus treatment in this patient exhibited its potential to treat recurrent PTPR among adults with *PTEN* and chromosome 10 loss, with implications for further consideration and investigation of the efficacy of everolimus as a possible novel treatment strategy. More studies on effective treatment strategies investigating the mTOR pathway for PTPR are needed. Further insight into the differentiating factors between ependymomas and PTPR may provide a better understanding of these tumors to facilitate quicker and more accurate diagnoses and pave the way for prognostic and therapeutic implications.

## Data Availability

The data presented in this study are available on request from the corresponding author. The data are not publicly available due to patient confidentiality pertaining to medical electronic health records from which our data were collected.
